# *OsWRKY97,* an Abiotic Stress-Induced Gene of Rice, Plays a Key Role in Drought Tolerance

**DOI:** 10.3390/plants12183338

**Published:** 2023-09-21

**Authors:** Miaomiao Lv, Dejia Hou, Jiale Wan, Taozhi Ye, Lin Zhang, Jiangbo Fan, Chunliu Li, Yilun Dong, Wenqian Chen, Songhao Rong, Yihao Sun, Jinghong Xu, Liangjun Cai, Xiaoling Gao, Jianqing Zhu, Zhengjian Huang, Zhengjun Xu, Lihua Li

**Affiliations:** 1 Rice Research Institute of Sichuan Agricultural University, Chengdu 611130, Chinarongsh@btmc.edu.cn (S.R.); phosphate@126.com (Z.H.); 2 College of Engineering, Huazhong Agricultural University, Wuhan 430070, China; hdj05@mail.hzau.edu.cn; 3 Crop Research Institute, Academy of Agricultural and Forestry Sciences, Chengdu 611130, China; 4 Crop Ecophysiology and Cultivation Key Laboratory of Sichuan Province, Chengdu 611130, China; 5 State Key Laboratory of Crop Gene Exploration and Utilization in Southwest China, Sichuan Agricultural University, Chengdu 611130, China

**Keywords:** drought tolerance, ABA, *OsWRKY97*, antioxidation, *Oryza sativa* L.

## Abstract

Drought stress is one of the major causes of crop losses. The WRKY families play important roles in the regulation of many plant processes, including drought stress response. However, the function of individual WRKY genes in plants is still under investigation. Here, we identified a new member of the WRKY families, *OsWRKY97*, and analyzed its role in stress resistance by using a series of transgenic plant lines. *OsWRKY97* positively regulates drought tolerance in rice. *OsWRKY97* was expressed in all examined tissues and could be induced by various abiotic stresses and abscisic acid (ABA). *OsWRKY97-GFP* was localized to the nucleus. Various abiotic stress-related cis-acting elements were observed in the promoters of *OsWRKY97*. The results of *OsWRKY97*-overexpressing plant analyses revealed that *OsWRKY97* plays a positive role in drought stress tolerance. In addition, physiological analyses revealed that *OsWRKY97* improves drought stress tolerance by improving the osmotic adjustment ability, oxidative stress tolerance, and water retention capacity of the plant. Furthermore, *OsWRKY97*-overexpressing plants also showed higher sensitivity to exogenous ABA compared with that of wild-type rice (WT). Overexpression of *OsWRKY97* also affected the transcript levels of ABA-responsive genes and the accumulation of ABA. These results indicate that *OsWRKY97* plays a crucial role in the response to drought stress and may possess high potential value in improving drought tolerance in rice.

## 1. Introduction

Drought is one of the major stresses that seriously affects plant growth and reduces yield [1]. Plants have evolved various strategies to cope with drought stress for survival and development. The response process of plants to drought stress includes stress signal perception, signal transduction and amplification, and adaptation at morphological, physiological, and molecular levels [2]. In these processes, many diverse stress-related proteins are expressed that enhance drought resistance via outputs such as osmotic adjustment, stomatal closure, and reactive oxygen species (ROS) scavenging [3].

ABA has been characterized extensively as an important plant hormone, which responds to drought stress by regulating stomatal closure and transpiration rate [4,5]. It has been reported that most water stress-inducing genes respond to treatment with exogenous ABA and relate to ABA signal transduction pathways [6]. ABA-mediated stomatal closure is usually accompanied by the production of H_2_O_2_ [7]. It was reported that H_2_O_2_ participated in ABA signal transduction in plant guard cells and triggered stomatal closure by activating Ca^2+^ channels on cell membranes [8,9]. It has also been reported that H_2_O_2_ can induce stomatal closure, which was independent of the ABA pathway [10]. However, under stress conditions, the content of H_2_O_2_ increased sharply in plant cells, which posed a threat to plants, but it is also thought that ROS is a signal for activation of defense pathways [11].

The WRKY protein has a highly conserved WRKY domain, containing an almost unchanged WRKYGQK sequence at the N-terminal, followed by a Cx_4–5_Cx_22–23_ HxH or Cx_7_Cx_23_ HxC zinc-finger motif [12]. Based on the number of WRKY domains and the types of zinc finger motifs, WRKY proteins can be divided into groups I-III. Group I WRKY contains two WRKY domains, Group II WRKY contains a WRKY domain, and the I-II groups all contain a Cx_4–5_Cx_22–23_ HxH zinc-finger motif. Group III WRKY contains a WRKY domain and a Cx_7_Cx_23_ HxC zinc-finger motif [13].

At present, studies have revealed the involvement of the WRKY families in plant responses to abiotic stresses. For example, overexpression of *TaWRKY2* reduced the water loss rate of transgenic wheat and enhanced its drought resistance [14]. Furthermore, many transcription factors have been reported to be involved in ABA-mediated signaling pathways of plant responses to drought stress [15]. *CmWRKY10* is located in the nucleus, and overexpression of *CmWRKY10* enhanced chrysanthemum tolerance to drought stress through the ABA-signaling pathway [16]. *OsWRKY45* plays an important role in drought tolerance and ABA signal regulation in rice [17]. AREB/ABF transcription factor needs post-translational modification to activate. Some studies show that AREB requires ABA for full activation, and its activity is regulated by ABA-dependent multi-site phosphorylation of the conserved domains. Further analysis shows that SnRK2s play a key role in ABA-dependent phosphorylation of AREB/ABF [18]. The default state of SnRK2 kinase is the active state of autophosphorylation. PP2Cs make SnRK2 kinase inactive by dephosphorylation [19]. In the presence of ABA, the PYR/PYL/RCARs can disrupt the interaction between the SnRK2s and PP2Cs, thus preventing the PP2C-mediated dephosphorylation of the SnRK2s and resulting in the activation of the SnRK2 kinases [20]. It has been reported that WRKY transcription factors regulate the expression of many genes related to ABA-induced physiology and development by binding the w-box sequence in the promoters of ABFs/AREBs [21]. *AtWRKY63* mutants impair the sensitivity of ABA-mediated stomatal closure and affect its drought tolerance [22]. It is reported that *WRKY18*, *WRKY60*, and *WRKY40* play an important role in the complex mechanism of ABA signal transduction. It is found that *WRKY40* and *WRKY60* transcription factors inhibit *ABI4* gene expression. In addition, their mutual antagonism not only occurs between *WRKY40* and *WRKY60*, but also between *WRKY18* and *WRKY40* to balance the repressive functions on the *ABI4* gene. However, for the *ABI5* gene, *WRKY60, WRKY18*, and *WRKY40* inhibit *ABI5* independently, but *WRKY60* also antagonizes the inhibition of *WRKY18* and *WRKY18-WRKY40* heterodimer to balance the inhibition of *ABI5* gene [23].

The corresponding sequence of *OsWRKY97* (LOC_Os12g02420.1) can be downloaded from the Rice Genome Annotation Project (RGAP) (http://rice.uga.edu/, accessed on 10 July 2019) and *OsWRKY97* belongs to the WRKY families. In this present research, we found that the expression level of *OsWRKY97* in wide-type rice was significantly improved under abiotic stress. And *OsWRKY97*-overexpressing plant analyses indicate that *OsWRKY97* positively regulates abiotic stress tolerance and ABA sensitivity. These findings contribute to our understanding of *OsWRKY97*-mediated drought responses and ABA signaling, and has potential application in genetically modified crops with improved drought tolerance.

## 2. Results

### 2.1. Expression Patterns of OsWRKY97

**Figure 1:**
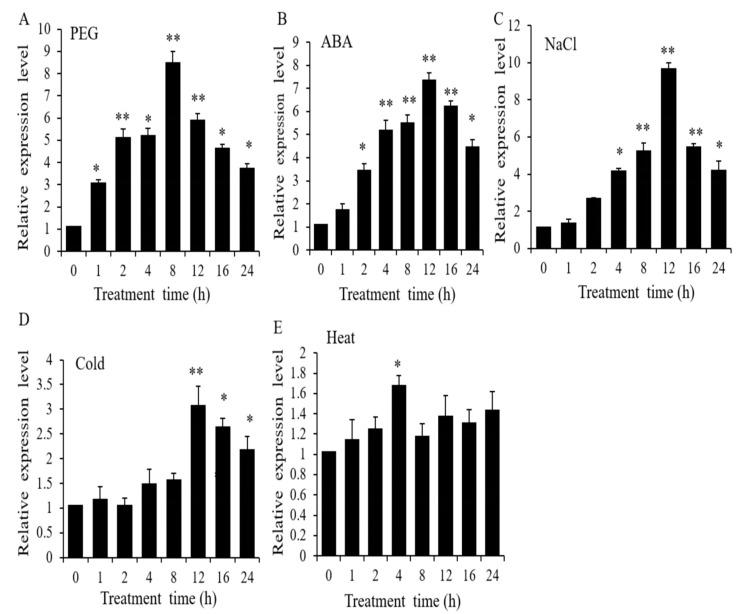
Expression analysis of the *OsWRKY97* gene in rice. (**A**–**E**) *OsWRKY97* expression analysis (qRT-PCR) in leaves of 2-week-old rice seedlings subjected to 20% (*w*/*v*) PEG6000, 50 µM ABA, 250 mM NaCl, cold (4 °C), and heat (42 °C) treatments, respectively. *OsActin* gene was used as internal controls. The data are represented as the mean ± SD (n = 3), with three biological experiments. Asterisks indicates a significant difference from the value at 0 h (*t*-test, * *p* < 0.05, ** *p* < 0.01).

We examined the expression of *OsWRKY97* in 15 different tissues by Real-Time Quantitative Polymerase Chain Reaction (qRT-PCR), so that the expression pattern of *OsWRKY97* in different tissues at different stages of rice was detected. *OsWRKY97* was expressed in various tissues of rice at different stages (Figure S1). We investigated the expression patterns of *OsWRKY97* seedlings under various abiotic stresses by qRT-PCR. These results suggested that *OsWRKY97* may be involved in responses to various stresses (Figure 1). For drought treatment, the transcription level of *OsWRKY97* gradually increased until reaching a maximum at 8 h (Figure 1A). The transcription level of *OsWRKY97* reached its maximum after treatment with 50 µM ABA for 12 h, and similar results were obtained after treatment with 250 mM NaCl (Figure 1B,C). The expression of *OsWRKY97* increased significantly in 12 h to 24 h under cold stress (Figure 1D). However, the transcription level of *OsWRKY97* under high-temperature treatment was not significantly induced (Figure 1E).

### 2.2. Phylogenetic Analysis of OsWRKY97

**Figure 2:**
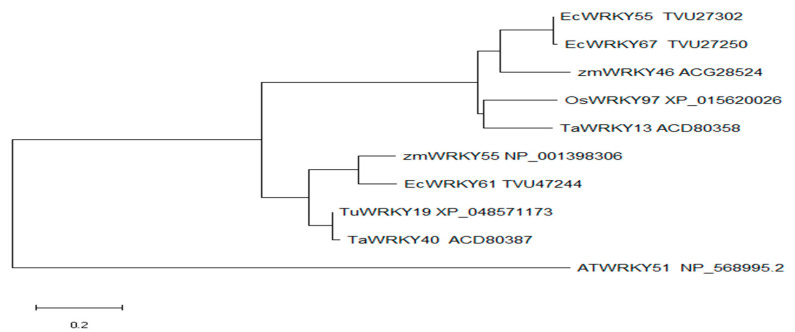
Phylogenetic tree of the protein sequences of *OsWRKY97* and other similar sequences.

We searched the homologous amino acid sequence of *OsWRKY97* by using the National Center for Biotechnology Information (NCBI, http://www.ncbi.nlm.nih.gov/, accessed on 10 July 2019) BLASTp online tool. The phylogenetic tree of OsWRKY97 protein sequences and other similar sequences was constructed using the N-J method in MEGA-X software. The results showed that *OsWRKY97* is closer to *TaWRKY13*, followed by *ZmWRKY46* (Figure 2). However, the identity of *OsWRKY97* with other orthologs was lower than that with *TaWRKY13*; this shows that *OsWRKY97* has a wide range of changes with other members.

### 2.3. Subcellular Localization of OsWRKY97

**Figure 3:**
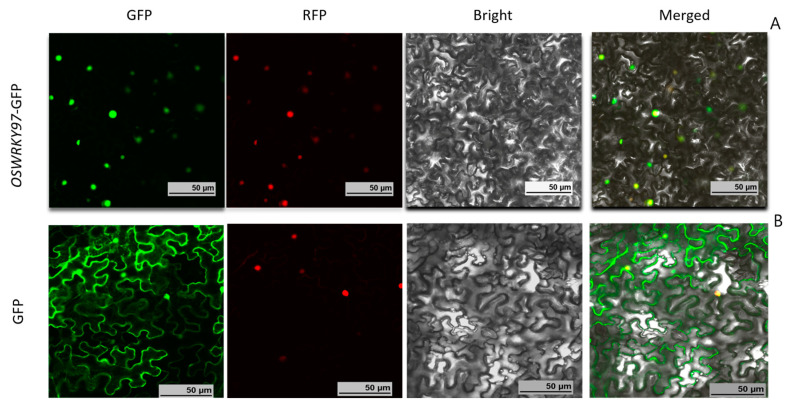
Subcellular localization of *OsWRKY97*-GFP. *OsWRKY97* driven by the CaMV35S promoter was transiently expressed in tobacco leaf epidermal cells and viewed with confocal microscopy. Nuclear and cytosolic localization of GFP protein was shown as a control. (**A**) 35S: *OsWRKY97*-GFP; (**B**) 35S: GFP. Bar = 50 µm.

To determine the subcellular localization of *OsWRKY97*, the full-length cDNA sequence of *OsWRKY97* was fused to green fluorescent protein (GFP) and driven by CaMV35S. The fusion protein and GFP control were transiently expressed in tobacco cells via *Agrobacterium* infiltration, and meanwhile, the carrier of the red fluorescent protein (RFP) NLS-RFP connected with the nuclear localization signal is used as the nuclear localization control. The fluorescence signal of the fusion protein was located in the nucleus (Figure 3A), while the fluorescence signal of the GFP control was located in the nucleus and cytoplasm (Figure 3B). *OsWRKY97*-GFP fusion protein and NLS-RFP were co-located in the nucleus, indicating that *OsWRKY97* is a nuclear protein.

### 2.4. Analysis of OsWRKY97 Promoter Domain

The promoter region contains many cis-acting elements related to stress response. In order to further understand the regulatory mechanism of *OsWRKY97*, we analyzed the promoter region upstream of *OsWRKY97* ATG initiation codon. The results showed that the promoter region contained the W-box elements, and MYB and MYC binding sequences. In addition, gibberellin-responsive elements (GAREs) and SA-responsive element (W-Box) were identified (Table 1).

### 2.5. Overexpression of OsWRKY97 in Rice Improved Osmotic Stress Tolerance at the Germination and Post-Germination Stages

**Figure 4:**
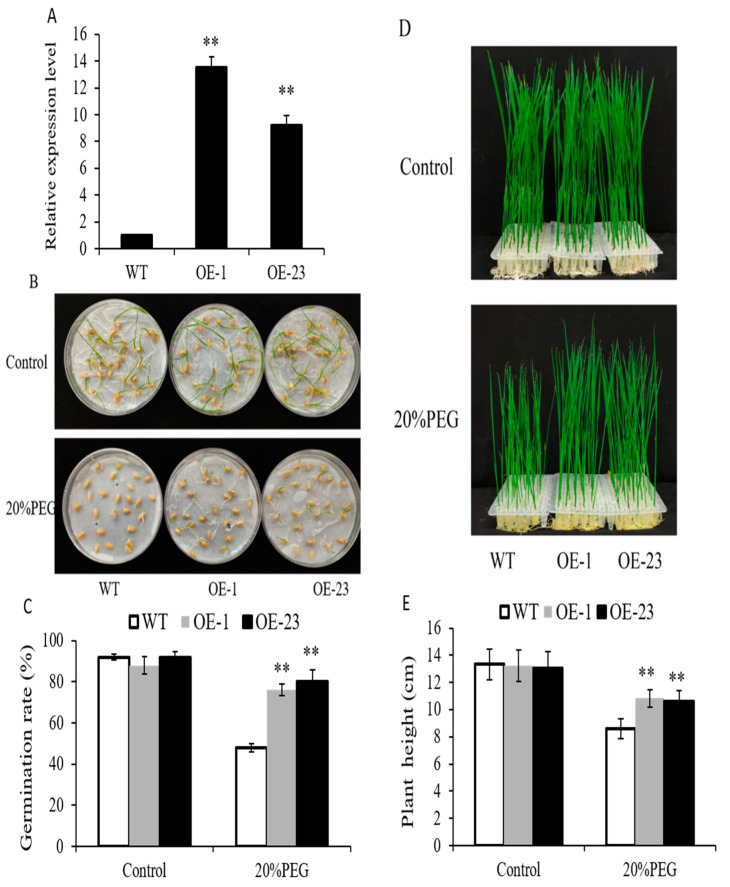
Phenotype of *OsWRKY97*-overexpressing plants under osmotic stress. (**A**) Two independent transgenic lines (OE-1, OE-23) of *OsWRKY97* were verified by qRT-PCR. *OsActin* was analyzed as an internal control. Data were means ± SD with at least three biological replicates. Asterisks represent statistically significant differences between WT and *OsWRKY97* overexpression lines (*t*-test, ** *p* < 0.01). The growth performance (**B**) and germination rate (**C**) of *OsWRKY97* overexpression lines and wild-type at 5 days after germination in nutrient solution under normal and osmotic stress conditions. The data are represented as the mean ± SD with at least three biological replicates, and every replicate contains 20 individual plants. The growth phenotype (**D**) and plant height © of overexpression lines and WT, which were growing under normal conditions for 4 days, were transferred to nutrient solution under normal and osmotic stress conditions for 12 days. (**E**) Data are shown as the mean ± SD (n = 4) with four biological replicates, and every replicate contains 40 individual plants. The asterisk represents the statistically significant difference between the WT and *OsWRKY97* overexpression lines under osmotic stress (*t*-test, ** *p* < 0.01).

In this study, we found that *OsWRKY97* was strongly induced by osmotic stress (Figure 1A). To further verify whether *OsWRKY97* was involved in regulating the sensitivity of rice to stress, we constructed an *OsWRKY97* overexpression vector and transferred it into wild-type rice. The expression level of *OsWRKY97* in overexpressed plants (OE) was analyzed by qRT-PCR. The results showed that the expression of *OsWRKY97* was significantly enhanced in transgenic lines (Figure 4A). The screened T2 transgenic plants all showed significant response to drought stress, but the OE-1 and OE-23 lines showed the most significant performance. Therefore, these two independent transgenic rice lines, OE-1 and OE-23, were selected for future testing. Under normal conditions, the germination rates of WT and overexpression lines were not significantly different. However, after 5 days of absorption under osmotic stress (20% (*w*/*v*) PEG6000), the germination rate of overexpressed lines OE-1 and OE-23 (76% and 80%, respectively) were higher than that of WT (50%) (Figure 4B,C). To learn about the sensitivity of overexpression lines to osmotic stress at the post-germination stage, seedings of WT and overexpression lines that were growing under normal conditions for 4 days were selected and transferred to the nutrient solution under normal and osmotic stress conditions. After 12 days, each material was photographed, and its plant height was measured. Under normal conditions, the seedlings of WT and overexpressing lines were similar in growth. Under osmotic stress, the plant height of WT seedlings was 8.6 cm, while the plant height of overexpression seeding OE-1 was 10.8 cm, and that of OE-23 was 10.6 cm (Figure 4D,E). These results showed that overexpression of *OsWRKY97* in rice did not affect seed germination and seedling growth under normal growth conditions, but significantly attenuated the inhibitory effects on seed germination and seedling under osmotic stress induced by 20% (*w*/*v*) PEG6000.

### 2.6. Overexpression of OsWRKY97 in Rice Enhanced Drought Stress Tolerance

**Figure 5:**
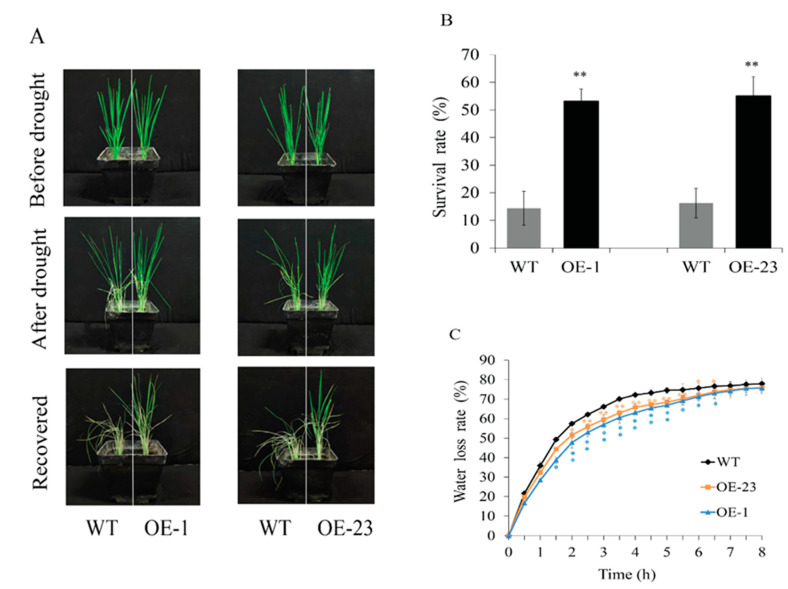
Drought stress analysis of wild-type plants and transgenic plants with *OsWRKY97*. (**A**) Performance of 2-week-old seedlings from *OsWRKY97* transgenic and wild-type plants subjected to drought stress without water for 15 days and then recovered for 3 days. The experiment contained three biological replicates. (**B**) Survival rates of transgenic and WT plants under drought stress. Values represent the mean ± SD (n = 3) from three independent biological experiments, and every replicate contains 12 individual plants. Statistically significant differences between WT and *OsWRKY97* overexpression lines were indicated by asterisks (*t*-test, ** *p* < 0.01). (**C**) Rate of water loss by detached leaves from control and transgenic plants. It is expressed as percentage of initial fresh weight. Values are the mean ± SD (n = 3) from three independent biological experiments, and every replicate contains 5 individual plants. The asterisk represents the statistically significant difference at the same time between the WT and *OsWRKY97* overexpression lines (*t*-test, ** *p* < 0.01).

To further validate the biological function of *OsWRKY97*, we tested the drought stress tolerance of *OsWRKY97* overexpression lines by water deficit. *OsWRKY97*-overexpressing lines and WT seedlings with similar vigor were sown in the same pots, watering was stopped until the leaves curled, and then resumed. Growth conditions were similar for all plants before stress was applied. After drought treatment, WT plants showed more severe leaf curling than *OsWRKY97*-overexpressing plants. After re-watering, the survival rate of overexpressed plant OE-1 was 53%, and that of OE-23 was 55%; however, the survival rate of WT was only 14–16% (Figure 5A,B). These results indicated that overexpression of *OsWRKY97* enhances the drought tolerance of rice. The relative water loss rate of detached leaves is an important characteristic reflecting drought tolerance [24]. Leaves of 2-week-old transgenic plants and WT seedlings were removed and exposed to water-free air for dehydration. Compared with WT, the leaf water loss of overexpression lines was significantly slower (Figure 5C), which means that *OsWRKY97* played an active role in improving the water retention capacity of plants under dehydration conditions.

### 2.7. Effects of OsWRKY97 Overexpression on Related Physiological Indexes under Drought Stress

**Figure 6:**
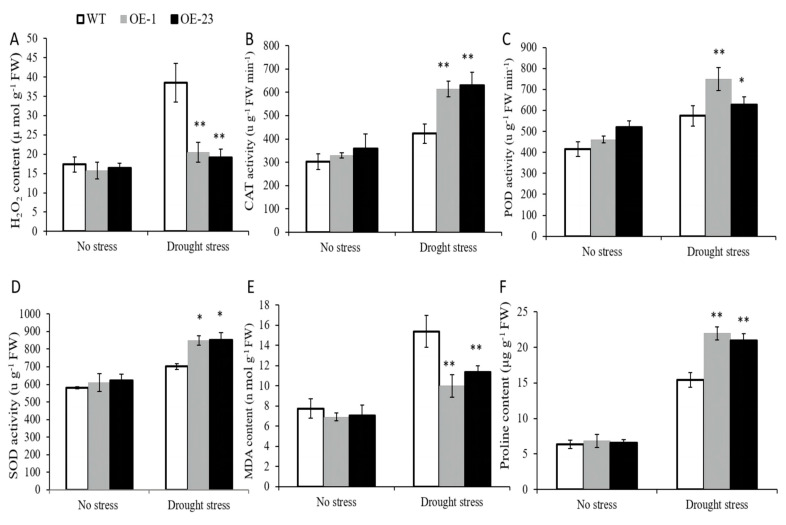
Physiological parameters of *OsWRKY97* overexpressed plants under drought stress. Two-week-old rice seedlings were transferred to nutrient solution under normal and drought stress conditions (20% (*w*/*v*) PEG6000). (**A**) H_2_O_2_ content, (**B**) CAT activity, (**C**) POD activity, (**D**) SOD activity, (**E**) MDA content, (**F**) proline content. Values are shown as the mean ± SD (n = 3) and the experiments were performed with at least three biological repetitions. The asterisk represents the statistically significant difference between the WT and OsWRKY97 overexpression lines under osmotic stress (*t*-test, * *p* < 0.05, ** *p* < 0.01).

To further clarify the physiological mechanism by which *OsWRKY97* confers tolerance to drought, we investigated the possible physiological basis related to the enhancement of drought resistance in *OsWRKY97*-overexpressing plants. ROS were generated in plants when they were subjected to abiotic stresses such as drought, salinity, heat, and cold [25]. H_2_O_2_, which is an important second messenger, is one of the most significant of these ROS [26]. Therefore, we measured the accumulation of H_2_O_2_ in plants after drought treatment. We found that the accumulation of H_2_O_2_ in *OsWRKY97*-overexpressing plants after drought treatment was much lower than that of WT (Figure 6A). To explore the potential mechanism of the reduction of active oxygen level in transgenic plants, ROS scavenging enzyme activity was also measured [11]. As shown in Figure 6, there was no significant difference in superoxide dismutase (SOD), catalase (CAT), and peroxidase (POD) activities between transgenic lines and WT under normal conditions, while the enzyme activities of transgenic lines were higher than those of WT under drought stress (Figure 6B,D). Abiotic stresses to plants lead to enhanced membrane peroxidation and accumulation of osmotic substances in plants to maintain osmotic potential tissues of leaves [27]. As shown in Figure 6E, under drought conditions, the contents of malondialdehyde (MDA) in the *OsWRKY97*-overexpressing plants were significantly lower than that in the WT, which indicated that overexpression of *OsWRKY97* in rice could reduce membrane lipid peroxidation under drought stress. Under drought stress, the proline content of transgenic plants was significantly higher than that of control plants (Figure 6F). This result clearly shows that overexpression of *OsWRKY97* can increase proline synthesis and protect rice plants to better cope with drought stress.

### 2.8. OsWRKY97 Is a Positive Regulator in ABA Signaling under Drought Stress

**Figure 7:**
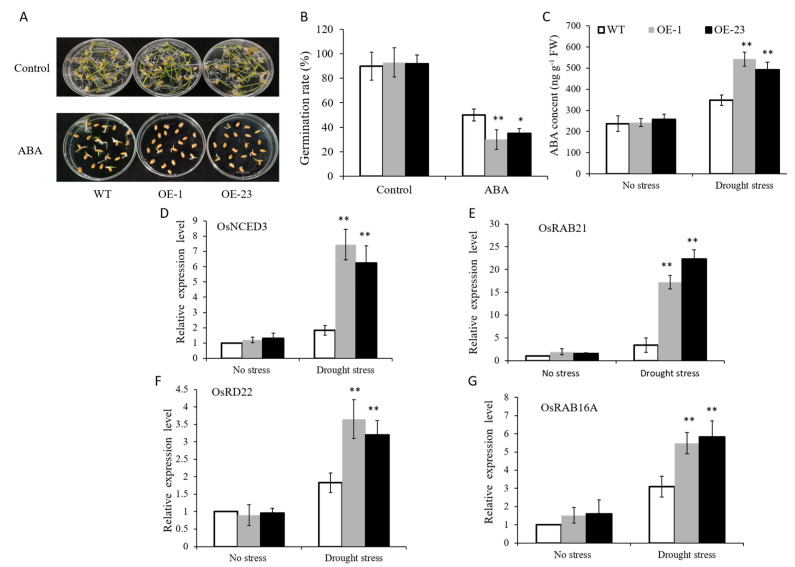
ABA accumulation and sensitivity of *OsWRKY97*-overexpressing plants. The growth performance (**A**) and the germination rates (**B**) of *OsWRKY97* overexpression and WT seeds under ABA treatment. Values are shown as the mean ± SD from three biological experiments, with 20 plants in each repeat. The asterisk represents the statistically significant difference between the WT and *OsWRKY97* overexpression lines under exogenous ABA stress (*t*-test, * *p* < 0.05, ** *p* < 0.01). (**C**) ABA contents of *OsWRKY97* overexpression and WT plants under normal and drought stress conditions. Results are means ± SD from three independent biological experiments. (**D**–**G**) Real-time PCR analysis of the expression of ABA biosynthesis and responsive genes under normal and drought stress conditions. The data are represented as the mean ± SD, with three biological experiments. The asterisk represents the statistically significant difference between the WT and *OsWRKY97* overexpression lines under osmotic stress (*t*-test, * *p* < 0.05, ** *p* < 0.01).

In this study, we found that *OsWRKY97* was also strongly induced by ABA (Figure 1B), to verify this expression pattern, the sensitivities of *OsWRKY97*-overexpressing plants to exogenous ABA were examined. The results showed that the germination rate of WT plants was higher than *OsWRKY97*-overexpressing plants under the treatment of exogenous ABA (Figure 7A,B). Therefore, we suspect that the drought tolerance of *OsWRKY97*-overexpressing transgenic lines may be related to ABA. In order to verify this hypothesis, we measured the ABA content in *OsWRKY97*-overexpressing lines and WT, respectively, under drought stress. The results showed that the endogenous ABA level was not significantly different between WT plants and *OsWRKY97*-overexpressing plants under normal conditions. However, under drought stress, the endogenous ABA level in *OsWRKY97*-overexpressing plants was significantly higher than WT plants (Figure 7C). In addition, we also analyzed the transcription level of response genes in the ABA signaling pathway in WT plants and *OsWRKY97*-overexpressing plants, including *OsRAB21*, *OsRD22*, *OsRAB16A*, and *OsNCED3* [28,29]. As shown in Figure 7D, the transcription level of these genes in *OsWRKY97*-overexpressing plants was significantly higher than WT plants under drought stress. These results indicate that *OsWRKY97* may improve drought tolerance via the ABA signaling pathway.

## 3. Discussion

Plants will suffer from various abiotic stresses in their natural environment, which will affect the normal development of plants and even affect their yield [30,31]. At present, many studies have shown that WRKY genes are negatively or positively involved in the integration of signaling pathways in abiotic stress responses [32]. However, the functions of many WRKY genes in plants, especially stress responses, are still unclear. In this study, we examined whether *OsWRKY97* participates in the regulation of the response to drought stress and its effect on rice. In addition, we found that *OsWRKY97*-GFP subcellular localization was in the nucleus of tobacco epidermal cells (Figure 3), which is consistent with previous studies on other WRKY families and may be related to its function [33].

Increasing evidence shows that WRKY families play an important role in the drought stress response, as ectopic expression of *OsWRKY11* enhanced tolerance to drought stress and induced constitutive expression of drought-responsive genes [34]. Overexpression of *GsWRKY20* from *Glycine soja* L.G07256 in *Arabidopsis* resulted in increased sensitivity to ABA when stomata were closed and stronger drought tolerance compared with the WT [35]. Similarly, our results showed that *OsWRKY97* expression was rapidly activated under drought stress, suggesting that *OsWRKY97* may play an important role in drought stress. Therefore, we constructed *OsWRKY97*-overexpressing transgenic plants and tested their resistance to drought stress. The results showed that the germination rate of *OsWRKY97*-overexpressing plants at the germination stage was significantly higher, and the growth inhibition of *OsWRKY97* at the seedling stage was also weakened compared with that of WT plants under osmotic stress (Figure 4). These results suggest that *OsWRKY97* may positively regulate drought stress tolerance. In addition, this conclusion was supported by the result that the survival rate of *OsWRKY97*-overexpressing plants was higher than that of WT plants under drought conditions (Figure 5A,B).

Plants respond to water loss at physiological, cellular, and molecular levels [36]. ABA is an important plant hormone involved in the plant developmental process, which is widely considered as the main regulatory factor of plant response to drought. ABA reduces water loss by inducing stomatal closure and induces a number of stress response genes [37,38]. It has been reported that ABA-independent and ABA-dependent regulatory systems both exist in response to drought stress [39]. In this study, first, we found that *OsWRKY97* was strongly induced by exogenous ABA (Figure 1B). Second, *OsWRKY97* overexpression enhances the sensitivity of plants to exogenous ABA (Figure 7C). Third, the expression level of *OsWRKY97* induces ABA accumulation and the expression level of ABA-responsive genes under drought stress (Figure 7A,D); this result was consistent with the capability of *OsWRKY97* to reduce the water loss rate of plants under drought conditions (Figure 5C). These results intimate that *OsWRKY97* improves drought tolerance by enhancing water retention of rice through the ABA-dependent pathway. Meanwhile, the up-regulation of *OsWRKY97* expression under drought stress will lead to the up-regulation of ABA biosynthesis and response genes, resulting in ABA accumulation and increased sensitivity to exogenous ABA.

H_2_O_2_ is not only an important ROS but also the pivot for the mutual conversion of ROS, which is also an important signal molecule at normal levels [26]. The ABA signal interacts with H_2_O_2_ in plant tissues. There is evidence that H_2_O_2_ acts upstream of the ABA signaling pathway. Exogenous H_2_O_2_ increases ABA catabolism during seed germination by enhancing the expression of *CYP707A* genes [40,41]. It has also been reported that H_2_O_2_ plays an important role as a second messenger in ABA-induced stomatal closure in guard cells [8,9]. It is reported that H_2_O_2_ can be induced by ABA, which is mediated by inducing plant gene expression encoding NADPH oxidase to respond to ABA [42,43]. However, the increase in H_2_O_2_ content induced by drought stress is more obvious than that caused by exogenous ABA; this shows that the water deficit signal enhances the production of ROS to a greater extent, which will pose a threat to plants [44]. As expected, the level of H_2_O_2_ in plants increased under drought stress. However, the accumulation of H_2_O_2_ in WT plants was significantly higher than that in *OsWRKY97*-overexpressing plants (Figure 6A), and the activities of major oxygen scavenging enzymes, including SOD, POD, and CAT, were also observably higher than those in *OsWRKY97*-overexpressed plants (Figure 6B,D). SOD, POD, and CAT, which maintain the ROS homeostasis, were activated in *OsWRKY97*-overexpressing plants.

As a product of ROS-stimulated lipid peroxidation, MDA contents can be used to evaluate the extent of ROS-mediated injuries in plants. In our research, we found that the accumulation of ROS and MDA in WT plants under drought stress were higher than that in *OsWRKY97*-overexpressing plants (Figure 6E). In addition, we also found that the accumulation of proline in *OsWRKY97*-overexpressing plants was greater than that in WT plants under drought stress (Figure 6F). Under osmotic conditions, proline, as an important osmotic protective agent, can maintain low water potential of cells so as not to be damaged by active enzymes [45]. These results showed that WT was more seriously damaged than *OsWRKY97*-overexpressing plants under osmotic conditions, thus increasing the probability of WT death.

## 4. Materials and Methods

### 4.1. Plant Material and Growth Conditions

The plant material *Oryza sativa* L. subsp. *japonica* cv. Nipponbare was used in this experiment as the wild-type rice. Rice seeds were sterilized with 0.1% NaClO for 30 min before being soaked in distilled water for 2 days in the dark and were then transferred to a culture dish containing *Hoagland* nutrient solution and grown in a climate chamber (Southeast instrument, Ningbo, China) with a temperature of 28 °C, a relative humidity of 70%, and a 14 h light/10 h dark photoperiod [46].

### 4.2. Abiotic Treatments

To determine the expression pattern of *OsWRKY97* under different stress conditions, two-week-old seedlings of Nipponbare rice were subjected to various stress treatments. For drought treatment, seedlings were grown in culture solution containing 20% (*w*/*v*) PEG6000. For salt treatment, NaCl solution was added to achieve a final concentration of 250 mM. ABA treatment was carried out by adding 50 µM ABA to the culture solution. The seedlings were transferred to a 4 °C climate chamber for cold treatment. For heat stress, the seedlings were subjected to 42 °C heat shock treatment [47,48]. Samples were collected at 0, 1, 2, 4, 8, 12, 16 and 24 h after treatment. Two-week-old seedings of *OsWRKY97* overexpression lines were subjected to drought treatment, and samples were collected at 0 h and 10 h after treatment, respectively.

### 4.3. RNA Extraction and Real-Time PCR

The leaves of 14-day-old plants were sampled, and total RNA was extracted with TRIzol reagent (Invitrogen, Nanjing, China) according to the manufacturer’s instructions. One microgram of DNase-treated RNA was reverse-transcribed using a RevertAid RT Reverse Transcription Kit (TaKaRa, Beijing, China) according to the manufacturer’s protocol. Real-Time Quantitative Polymerase Chain Reaction was performed on a Bio-Rad CFX96 real-time PCR system. Each reaction was performed in triplicate, and the reaction procedure was as follows: 95 °C for 10 min and, then 39 cycles of 95 °C for 10 s and, 60 °C for 30 s. The data of relative expression level were analyzed by the 2^−ΔΔCt^ method [49]. The *OsActin* rice gene was used as an internal control gene, and relevant primer pairs are listed in Table S1.

### 4.4. Sequence Analysis of OsWRKY97

The BLASTp online tool is used to search for homologous protein sequences of different dicot species in NCBI protein database. MEAG-X software was used to construct phylogenetic trees and neighbor-joining method with 1000 bootstrap was applied. The promoter region of 1.5 KB upstream of *OsWRKY97* gene was obtained from NCBI database, and the putative cis-acting elements of the promoter region were analyzed by PLACE database.

### 4.5. Vector Construction and Gene Transformation

To construct the overexpression vector of *OsWRKY97*, cDNA from rice (*Oryza sativa* L. *japonica* cv. Nipponbare) was used. Total RNA was used to amplify the open reading frame of *OsWRKY97*, and the relevant primer pairs are listed in Table S1. The fragments confirmed by sequencing were digested and cloned into the pCAMBIA1300 vector, and the digestion sites were HindΙΙΙ and SalΙ. This gene was driven by the CaMV35S promoter (Figure S2). All the vectors were transferred into Nipponbare plant calli via Agrobacterium-mediated transformation [50]. After producing the transgenic plants, the second generation of transgenic plants (T2) were screened using hygromycin and PCR detection.

### 4.6. Determination of Stress-Associated Physiological Indicators

Seedlings growing under normal conditions for two weeks were transferred to a solution under normal and osmotic stress conditions 20% (*w*/*v*) PEG6000. Samples were collected at 0 h and 10 h, and then the corresponding physiological indexes were measured. MDA content was qualified by thiobarbituric acid reaction method, as described by Gao [51]. H_2_O_2_ content was determined according to the method of Zhan [52]. For the water loss rate, two-week-old seedlings were selected, exposed to air under the condition of no water supply, and weighed and recorded every half hour until the weight was constant. Water loss rate was calculated by comparing the measured weight from each indicated time with the measurement at time zero. It is expressed as percentage of initial fresh weight [53]. Proline was detected by following the reported methods [54]. The activity of active oxygen-scavenging enzymes was determined according to previous methods [55].

### 4.7. Measurement of ABA Content

We analyzed the content of ABA in rice by liquid chromatography–mass spectrometry (LC-MS) [56]. Briefly, 1 g of frozen leaf tissue was extracted in 10 mL of acetone/water/acetic acid (80:19:1, *v*/*v*). The complete homogenate was incubated overnight in darkness at 4 °C. After that, they were vortexed and centrifuged at 15,000 rpm, 4 °C for 10 min, and the crude extract supernatant was collected and dried in in a rotavapor until an aqueous fractions were obtained. Dried samples were resuspended in 1 mL of acetonitrile/water/acetic acid (90:10:0.05, *v*/*v*), and filtered through a 0.45 µm PTFE filter (JET BIOFIL, Guangzhou, China). Quantification was performed by the standard addition method by spiking control plant samples with ABA solutions.

### 4.8. Subcellular Localization of OsWRKY97

The coding region of *OsWRKY97* was amplified and cloned into the pAcGFP1-N1 vector to generate the *OsWRKY97*-GFP (green fluorescent protein) fusion construct, which was inserted into the pCAMBIA1300 vector, and the constructed vector was transferred into *Agrobacterium*. After sequencing confirmation, the fusion structure and the control vector were co-transfected with another NLS-RFP vector, respectively, into tobacco cells by *Agrobacterium*-mediated transient expression method, and the fluorescence signal was observed by confocal laser scanning microscope [57].

### 4.9. Analysis of Stress Tolerance

Two independent T2 overexpression lines and WT seeds were soaked in clear water for two days after being sterilized by 0.1% NaClO, and then the seeds were transferred to culture solution for growth. When the plant height was approximately 1 cm, seedlings with similar growth potential were selected and transferred to nutrient solution containing 20% (*w*/*v*) PEG6000, and their growth conditions were observed and recorded. To simulate an arid environment, the two-week-old seedlings were transferred to the sand, and watering was stopped after two days of normal irrigation. The mortality rates were determined. In order to test the sensitivity of ABA sensitivity at germination stage, seeds of *OsWRKY97*-overexpressing plants and WT plants were germinated on culture solution containing 5 µM ABA, and the germination rate was calculated on the 6th day after germination.

### 4.10. Statistical Analysis

Results are reported as the mean ± standard deviation (SD) values of the three independent biological experiments; all experiments were repeated at least three times, Statistical analysis was performed using the Student’s *t*-test by SPSS 27.0 (IBM SPSS, Chicago, IL, USA) software package, * *p* < 0.05, ** *p* < 0.01.

## 5. Conclusions

In conclusion, we identified a nuclear gene *OsWRKY97*, which affects the sensitivity of rice to exogenous ABA and the accumulation of ABA content. In addition, *OsWRKY97* affects the redox balance and drought resistance of rice. Redox-related mechanisms might be involved in *OsWRKY97*-mediated drought tolerance, which might affect the content of proline and MDA in rice. All these results indicated that the *OsWRKY97* gene has high potential for improving rice drought resistance.

## Figures and Tables

**Table 1:** Putative cis-elements in the *OsWRKY97* promoter.

Elements	Sequence	Function
MYB	WAACCA/YAACKG/CTAACCA/CNGTTR/AACGG/TAACAAA/TAACAAA/MACCWAMC/CCWACC/GGATA	ABA and drought-responsive elements
CGTCA-motif	CGTCA	Involved the MeJA-responsiveness
MYC	CATGTG/CANNTG	ABA and drought-responsive elements
GARE	TAACAA	GA-responsive element
W-Box	TTTGACY/TTGAC/CTGACY/TGACY	SA-responsive element
TC-rich repeats	GTTTTCTTAC	involved in defense and stress-responsiveness
TCA-element	CCATCTTTTT	involved in SA-responsiveness

## Data Availability

The *OsHis1.1* gene information are available in the National Center for Biotechnology Information (NCBI) repository (XP_015620026.1). The datasets supporting the findings of this article are included within the article and its additional files. The data are available from the corresponding author on reasonable request.

## Supplementary Materials

The following supporting information can be downloaded at: https://www.mdpi.com/article/10.3390/plants12183338/s1, Figure S1: Real time PCR analysis of OsWRKY97 gene in rice Nipponbare different tissues, Figure S2: Schematic diagram of the architecture of the vector; Table S1: Primer sequences used in this study.
