# Supplementary material for: OsWRKY97, an Abiotic Stress-Induced Gene of Rice, Plays a Key Role in Drought Tolerance
Source: Plants (Basel). 2023 Sep 21;12(18):3338. doi: 10.3390/plants12183338 (PMC10536077; doi:10.3390/plants12183338)
Supplement: Supplementary file 1 [file plants-12-03338-s001.zip › plants-2602518-supplementary.pdf]

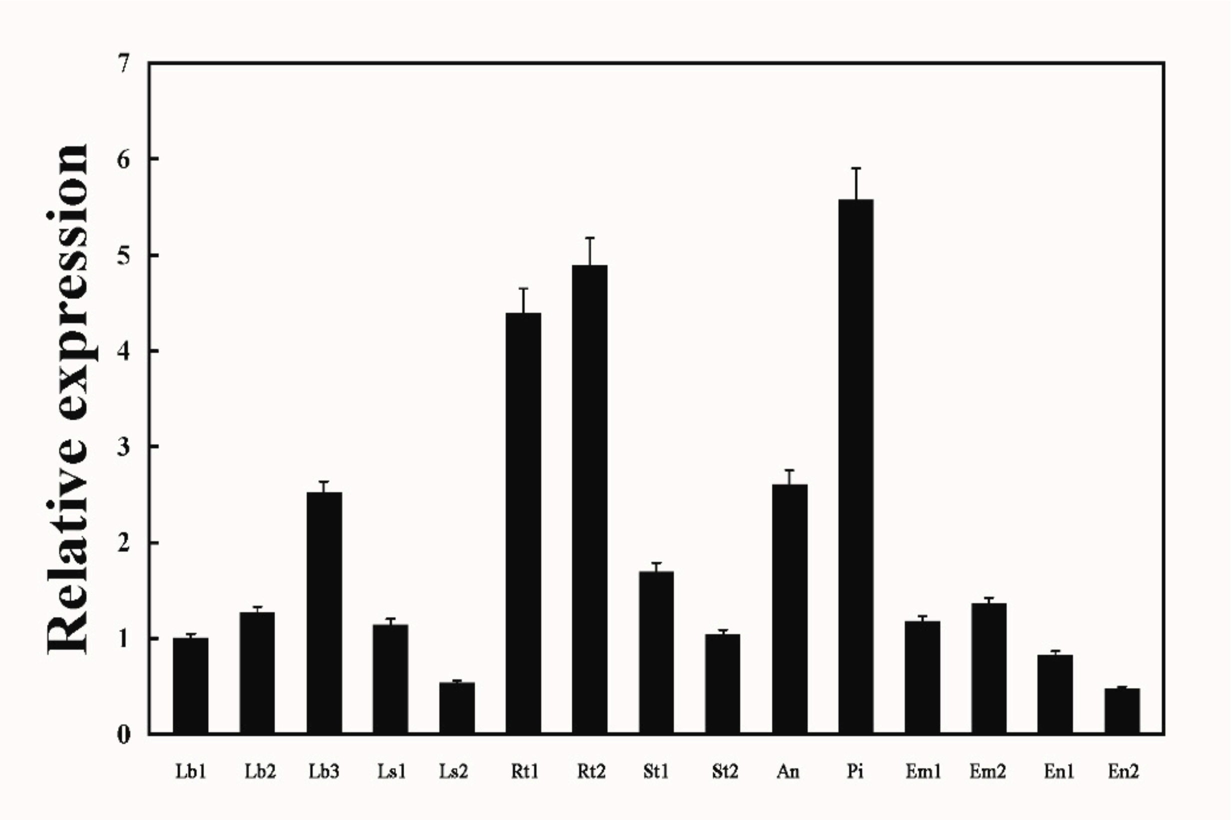

Supplementary Figure S1. Real time PCR analysis of OsWRKY97 gene in rice Nipponbare different tissues. Fifteen representative tissues are as follows: Lb1, leaf blade at four-leaf stage; Lb2, leaf blade from plants with four tillers; Lb3, leaf blade at ripening stage; Ls1, Leaf sheath at four-leaf stages; Ls2, Leaf sheath from plants with four tillers; Rt1, root at four-leaf stages; Rt2, root at from plants with four tillers; St1, stem from plants with four tillers; St2, stem at ripening stage; An, 1.2-1.5 mm anther; Pi, pistil from 10-14 cm inflorescence; Em1, embryo at 7 days after flowering; Em2, embryo at 28 days after flowering; En1, endosperm at 7 days after flowering; En2, endosperm at 28 days after flowering. The rice Actin transcript levels were used as internal controls. Error bars indicate SE based on three biological replicates.

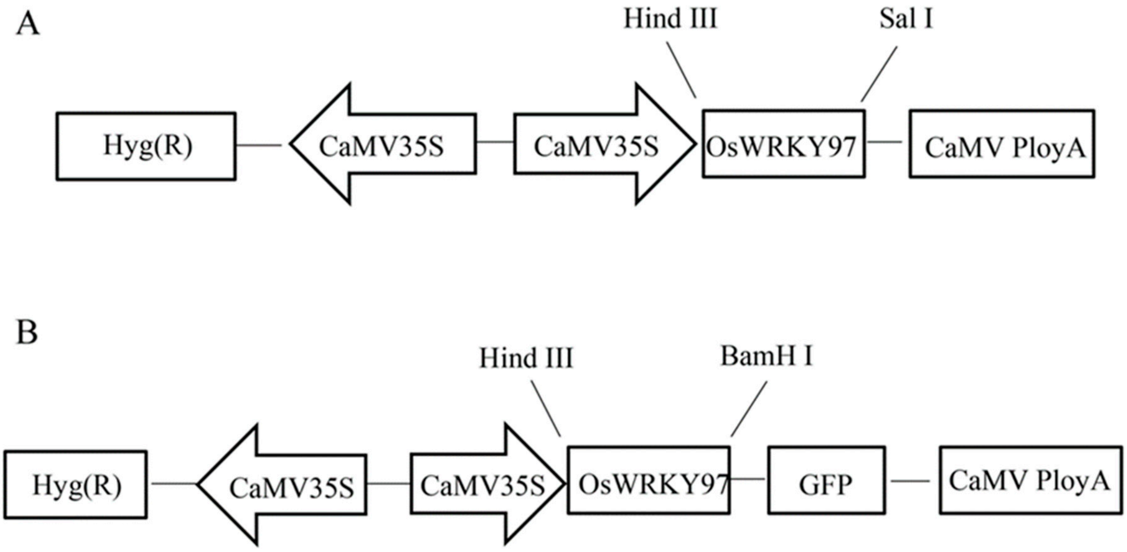

Supplementary Figure S2. Schematic diagram of the architecture of the vector. (A) schematic diagram of the architecture of the OsWRKY97 overexpression. (B) schematic diagram of the architecture of the OsWRKY97-GFP

Table S1 Primer sequences used in this study

Primer name	Primer sequence (5'-3')
OsWRKY97 for clone	Forward: AAGCTTTCCCTACCAAGCGAGACCA<br>Reverse: GTCGACCCCTATCTGGCACCATCCA
OsWRKY97-GFP for clone	Forward: AAGCTTCCTACCAAGCGAGACCACT<br>Reverse: AGATCTCAAGAAATGGAAATACTCG
OsWRKY97 for qRT-PCR	Forward: AGCCGTACAGTTCCAACCAG<br>Reverse: AATGTCCTCGACAGAGCACC
OsActin for qRT -PCR	Forward: ACAACTGGGACGACATGGAG<br>Reverse: GCCACATACATTGCTGGTG
OsRAB21 for qRT-PCR	Forward: CAGTTCCAGCCGATGAGGG<br>Reverse: CTGCTGCTCGCCCTTGTT
OsRD22 for qRT-PCR	Forward: CTAGTCTCTCGCTGCTCTCCT<br>Reverse: GCGCAGTAGTGCTTGTGCTTG
OsRAB16A for qRT-PCR	Forward: GCCGAGTAACTGGGGTCAAG<br>Reverse: GTTTCTTGGCTGGTGTGCTC
OsNCED3 for qRT-PCR	Forward: AGTACTTCTACTTCGCGCCC<br>Reverse: CGGGTACCACCACGTAGTTC
